# Dual Role for Astroglial Copper-Assisted Polyamine Metabolism during Intense Network Activity

**DOI:** 10.3390/biom11040604

**Published:** 2021-04-19

**Authors:** Zsolt Szabó, Márton Péter, László Héja, Julianna Kardos

**Affiliations:** 1 Functional Pharmacology Research Group, Research Centre for Natural Sciences, Institute of Organic Chemistry, H-1117 Budapest, Hungary; szabo.zsolt@ttk.hu (Z.S.); peter.marton@ttk.hu (M.P.); kardos.julianna@ttk.hu (J.K.); 2 Hevesy György Ph.D. School of Chemistry, ELTE Eötvös Loránd University, H-1117 Budapest, Hungary

**Keywords:** astroglial tonic inhibitory feedback, astroglial GABA transporter, copper transporter CTR1, putrescine catabolism, copper amino oxidase, Cx43 gap junction channel

## Abstract

Astrocytes serve essential roles in human brain function and diseases. Growing evidence indicates that astrocytes are central players of the feedback modulation of excitatory Glu signalling during epileptiform activity via Glu-GABA exchange. The underlying mechanism results in the increase of tonic inhibition by reverse operation of the astroglial GABA transporter, induced by Glu-Na^+^ symport. GABA, released from astrocytes, is synthesized from the polyamine (PA) putrescine and this process involves copper amino oxidase. Through this pathway, putrescine can be considered as an important source of inhibitory signaling that counterbalances epileptic discharges. Putrescine, however, is also a precursor for spermine that is known to enhance gap junction channel communication and, consequently, supports long-range Ca^2+^ signaling and contributes to spreading of excitatory activity through the astrocytic syncytium. Recently, we presented the possibility of neuron-glia redox coupling through copper (Cu^+^/Cu^2+^) signaling and oxidative putrescine catabolism. In the current work, we explore whether the Cu^+^/Cu^2+^ homeostasis is involved in astrocytic control on neuronal excitability by regulating PA catabolism. We provide supporting experimental data underlying this hypothesis. We show that the blockade of copper transporter (CTR1) by AgNO_3_ (3.6 µM) prevents GABA transporter-mediated tonic inhibitory currents, indicating causal relationship between copper (Cu^+^/Cu^2+^) uptake and the catabolism of putrescine to GABA in astrocytes. In addition, we show that MnCl_2_ (20 μM), an inhibitor of the divalent metal transporter DMT1, also prevents the astrocytic Glu-GABA exchange. Furthermore, we observed that facilitation of copper uptake by added CuCl_2_ (2 µM) boosts tonic inhibitory currents. These findings corroborate the hypothesis that modulation of neuron-glia coupling by copper uptake drives putrescine → GABA transformation, which leads to subsequent Glu-GABA exchange and tonic inhibition. Findings may in turn highlight the potential role of copper signaling in fine-tuning the activity of the tripartite synapse.

## 1. Introduction

Several lines of evidence along with theoretical considerations discuss the potential of neuron-glia coupling in healthy and diseased brain [1,2,3,4,5,6,7,8]. Beside neuronal activity-dependent astroglial energy metabolisms [9], neuron-glia coupling may involve K^+^, Ca^2+^ and Na^+^ signalling through gap junction channels (GJCs), astroglial Glu-Na^+^ symport-evoked release of GABA (Glu-GABA exchange), glycine, glutamine and other neuro/glio transmitters or modulators [10,11,12,13,14,15,16,17,18,19,20,21,22,23,24]. In particular, astroglial GABA release mechanisms involve inside-out (reverse) operation of astroglial GABA transporters GAT-2/3 [15,25,26] or bestrophin 1 channels [27,28,29]. Nevertheless, GABA may travel far away from the tripartite synapse through astrocytic Cx43 GJCs, also facilitated by polyamines (PAs) [30,31].

Central inhibition is controlled by the major inhibitory neurotransmitter GABA that is mainly formed by decarboxylation of Glu in neurons. In midway dopaminergic neurons, however, it is formed from putrescine by the copper amino oxidase (CAO) and aldehyde dehydrogenase 1a1 enzymes [32]. Several lines of evidence suggest that the catabolism of putrescine to GABA in astrocytes [33,34] is the dominant source of the gliotransmitter GABA [15,26,28,35,36,37,38]. On the molecular level, the cofactor 2,4,5-trihydroxyphenylalanine quinone (topaquinone; TPQ), present in the D4 catalytic centre of mammalian CAOs is featured by three conserved hystidines coordinating the copper ion involved in TPQ biogenesis [39]. It is conceivable therefore, that GABA formation from putrescine [38] may in turn be reliant on copper (Cu^+^/Cu^2+^) homeostasis.

As an alternative to neuronal source-target-physiology scheme of copper signaling [40], we hypothesized a gliocentric scheme of redox signaling mediated by Cu^+^/Cu^2+^ [41]. The scheme suggests that copper released from depolarized nerve endings [42] and taken up primarily by the high-affinity copper transporter (CTR1) [43,44] may enhance the copper-catalyzed oxidative putrescine → GABA transformation, boosting astroglial Glu-GABA exchange, thereby tonic inhibition.

In order to assess the potential role for CTR1 in putrescine metabolism and Glu-GABA exchange, here we explored whether inhibition of CTR1 by Ag^+^ [45] or inhibition of the divalent metal transporter DMT1 by Mn^2+^ [46] affect the Glu-GABA exchange-controlled tonic inhibition in rat hippocampal slices. We hypothesized that lowering astroglial Cu^+^/Cu^2+^ level by CTR1 blockade may disrupt the ornithine → putrescine → GABA catabolism pathway [47] and consequently reduce the tonic inhibition mediated by the astrocytic Glu-GABA exchange. CTR1 blockade, however, may also diminish the putrescine → spermidine → spermine transform [48] via reduction of astroglial Cx43 GJC signaling [30,31]. The actual redox potential dependent balance of inhibition and excitation may place putrescine formation and metabolism in the centre of molecular mechanisms underlying many cellular functions of PAs [49,50,51,52,53,54,55,56,57,58,59,60,61,62,63]. This might have repercussions on pharmacoresistant epilepsy [64,65] or tubular sclerosis seizures [66] or memory [67,68].

## 2. Materials and Methods

### 2.1. Animals

Animals were kept and used in accordance with standard ethical guidelines and approved by the local Animal Care Committee, the Government Office for Pest County (Reference Nos. PEI/001/3671-4/2015 and PE/EA/3840-4/2016), the Hungarian Act of Animal Care and Experimentation (1998, XXVIII, section 243), European Communities Council Directive 24 November, 1986 (86/609/EEC) and EU Directive 2010/63/EU on the use and treatment of animals in experimental laboratories. All efforts were made to reduce animal suffering and the number of animals used.

### 2.2. Buffers

Buffers contained in mM ACSF: 129 NaCl, 5 KCl, 1.6 CaCl_2_, 1.8 MgSO_4_, 1.25 NaH_2_PO_4_, 21 NaHCO_3_, 10 glucose (pH 7.4); nominally Mg^2+^-free ACSF was prepared as control ACSF with no added Mg^2+^ (we estimated the Mg^2+^ concentration of this buffer to be approximately 1 μM). Pharmacological assessment was done by the following compounds: MnCl_2_ (20 μM), AgNO_3_ (3.6 μM), CuCl_2_ (2 μM) – all purchased from Sigma-Aldrich, Schnelldorf, Germany, SNAP-5114 (100 μM, Tocris, Bristol, UK).

### 2.3. Slice Preparation

Transverse, 300 μm thick hippocampal-entorhinal slices from 12- to 15-day-old Wistar rats (Toxicoop, Budapest, Hungary) were prepared in modified ACSF (75 mM sucrose, 87 mM NaCl, 2.5 mM KCl, 1.25 mM NaH_2_PO_4_, 7 mM MgSO_4_, 0.5 mM CaCl_2_, 25 mM NaHCO_3_, 25 mM glucose, continuously bubbled with 95% O_2_ + 5% CO_2_ gas mixture) at 4 °C. Slices were incubated in an interface-type chamber that was continuously circulated with ACSF for one hour at 37 °C (followed by incubation at room temperature) before performing the experiments. 

### 2.4. In Vitro Electrophysiology

Electrophysiological recordings were performed at 31 °C. Signals were recorded with Multiclamp700A amplifiers (Axon Instruments, Foster City, CA, USA), low-pass filtered at 2 kHz and digitized at 20 kHz (Digidata1320A, Axon Instruments). For single cell recording CA1 pyramidal cells were identified visually. Pipettes (4 to 5 MΩ) were filled with a solution containing (in mM) 130 CsMeSO_3_, 10 NaCl, 0.05 CaCl_2_, 2 ATP (magnesium salt), 1 EGTA and 10 HEPES (pH set to 7.3 with 1N CsOH). To suppress escape action currents 5 mM QX 314 (Tocris, Bristol, UK) was added. Cells were voltage-clamped at 0 mV (corrected for a calculated junction potential of +15 mV) to record GABA*ergic* (outward) currents. Input resistance was 150.7 ± 14.0 MΩ. If signs of seal deterioration or cell closure occurred (>20% change in the access resistance) the recordings were discarded. Synaptic recordings were made for 10 to 20 min in control conditions following 10 to 20 min of 100 μM SNAP-5114 application and 10 to 20 min washout. 

### 2.5. Data Evaluation

Holding currents were determined as previously described [26]. All-point histograms were plotted for each 1 s period of experimental traces. A Gaussian was fitted to the unskewed part of the histogram and the position of the center of the fitted Gaussian was used as the holding current. Values during SLEs were not included in data evaluation.

Spontaneous IPSCs were analyzed by a custom MATLAB script, based on the detection method of the MiniAnalysis software (Synaptosoft, Decatur, GA, USA), using 10 pA as amplitude threshold. IPSCs with event frequency values greater than 300 Hz were excluded to avoid duplicate IPSC detection.

Unless stated otherwise data are expressed as means ± S.E.M. and were analyzed using Student’s paired t-test or one-way analysis of variances with Bonferroni post hoc tests (OriginPro 8.0). A value of *p* < 0.05 was considered significant.

## 3. Results 

### 3.1. Assessing Astroglial GABA Transporter-Specific Component of Tonic Inhibitory Current in the Low-[Mg^2+^] Model of Experimental Epilepsy 

To explore whether copper uptake plays a role in putrescine-dependent tonic inhibition provided by astrocytes [25,26], we measured inhibitory currents on hippocampal CA1 neurons. Rat hippocampal slices were exposed to low-[Mg^2+^] ACSF which enhances excitatory activity and triggers the astrocytic Glu/GABA exchange mechanism [25,26]. This mechanism is mediated by the astrocytic GAT-2/3 GABA transporters that are reversed following increased Glu uptake and elevated intracellular GABA level. We blocked GAT-2/3 transporters by their specific, non-transportable inhibitor SNAP-5114 (100 µM) to determine the GAT-2/3 mediated component of the tonic current measured on CA1 neurons. CsMeSO_3_-based pipette solution (with added QX 314) was used to isolate GABA*ergic* (outward directed) currents in voltage clamped configuration by applying 0 mV holding potential.

**Figure 1:**
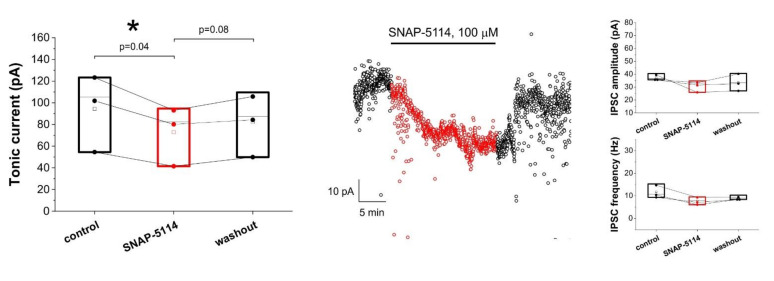
Glial GABA transporters release GABA and generate tonic inhibition in low-[Mg^2+^] ACSF. *Left:* Box-chart representation of GABA*ergic* baselines during control condition, in the presence of SNAP-5114 and washout. Box edges represent 25th, 50th and 75th percentile, open squares represent means, circles connected by lines represent paired individual baseline values. (N = 3). *Middle:* baseline currents plotted at 1 s intervals in a selected experiment. *Right*: amplitude and frequency of inhibitory postsynaptic currents (IPSCs). Box edges represent 25th, 50th and 75th percentile, open squares represent means, circles connected by lines represent paired individual baseline values. (N = 3). Neither amplitude, nor frequency changed significantly due to SNAP-5114 application (*p* = 0.31 and *p* = 0.29, respectively). Black symbols represent control and washout periods, red symbols represent SNAP-5114 application in all figures.

Under control condition, in low-[Mg^2+^] ACSF we observed that the blockade of GAT-2/3 transporters by 100 μM SNAP-5114 decreased the tonic current from 94.4 ± 20.6 pA to 72.8 ± 16.1 pA (Figure 1). The decrease was found to be significant (*p* = 0.04, N = 3). The GABA*ergic* origin of the measured current has previously been validated, since SNAP-5114 had no effect on the tonic current in the presence of the GABA_A_ antagonist picrotoxin [26]. The observed decrease in the tonic current is not due to a change in IPSC kinetics, since neither the amplitude, nor the frequency of IPSCs were altered significantly in the presence of SNAP-5114 (Figure 1).

### 3.2. Effects of Added AgNO_3_ or MnCl_2_ on the GAT-2/3 Specific Tonic Inhibitory Component

**Figure 2:**
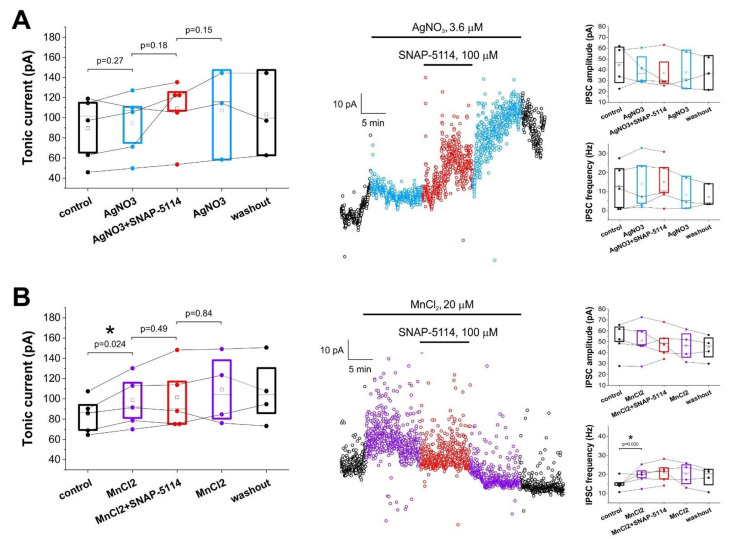
Added AgNO_3_ or MnCl_2_ prevents GABA release through glial GABA transporters in low-[Mg^2+^] ACSF. Effect of GAT-2/3 blockade by 100 µM SNAP-5114 on the holding current of voltage clamp recording segments in low-[Mg^2+^] ACSF in the presence of of 3.6 µM AgNO_3_ (**A**) or 20 μM MnCl_2_ (**B**). Left: Box-chart representation of GABA*ergic* baselines during control condition, AgNO_3_ or MnCl_2_ application, addition of SNAP-5114 and washout. Box edges represent 25th, 50th and 75th percentile, open squares represent means, circles connected by lines represent paired individual baseline values. (N = 5 for both AgNO_3_ and MnCl_2_). *Middle:* baseline currents plotted at 1 s intervals in a selected experiment. *Right*: amplitude and frequency of inhibitory postsynaptic currents (IPSCs). Box edges represent 25th, 50th and 75th percentile, open squares represent means, circles connected by lines represent paired individual baseline values. (N = 5 for both AgNO_3_ and MnCl_2_). Neither amplitude, nor frequency changed significantly in the presence of AgNO_3_ (*p* = 0.31 and *p* = 0.29, respectively), however MnCl_2_ significantly increased the frequency of IPSCs (*p* = 0.035). Black symbols represent control and washout periods, light blue and purple symbols represent AgNO_3_ or MnCl_2_ applications, respectively, red symbols represent simultaneous SNAP-5114 and AgNO_3_ or MnCl_2_ application in all figures.

In contrast to control conditions, SNAP-5114 did not significantly affect tonic currents when we blocked astrocytic copper uptake beforehand. Applying the copper transporter (CTR1) inhibitor AgNO_3_ (3.6 µM), the GAT-2/3 mediated tonic current disappeared and GAT-2/3 blockade even produced an intermediate, non-significant increase of baseline current (94.7 ± 14.4 pA in the absence vs. 109.4 ± 14.8 pA in the presence of SNAP-5114, *p* = 0.18, N = 5) (Figure 2A), suggesting that GAT-2/3 transporters operate in the normal mode, taking up GABA from the extracellular space when astrocytic GABA synthesis is impaired due to copper shortage. Surprisingly, addition of 20 μM MnCl_2_, a supposed inhibitor of another class of astrocytic copper transporters, the divalent metal transporter 1 (DMT1), resulted in the increase of tonic current from 84.9 ± 8.3 pA to 98.7 ± 11.6 pA (*p* = 0.024, N = 5), which was not significantly altered by the presence of SNAP-5114 (101.6 ± 14.3 pA in the presence of SNAP-5114, *p* = 0.49, N = 5) (Figure 2B). The increased tonic current in the presence of MnCl_2_, however, may be due to an alternative effect of Mn^2+^, since MnCl_2_ also increased IPSC frequency (Figure 2B). It is to note in this regard that Mn^2+^ is known to increase extracellular GABA concentration by altering the expression level of GAT-1 [69], but on the timescale of our experiments this is expected to have a negligible effect.

These results suggest that the suppression of copper entry into astrocytes by Ag^+^ leads to decreased GABA formation and consequently prevents GABA release through GAT-2/3 when it is operating in the reverse mode.

### 3.3. Direct Copper Application Generates Tonic Current

**Figure 3:**
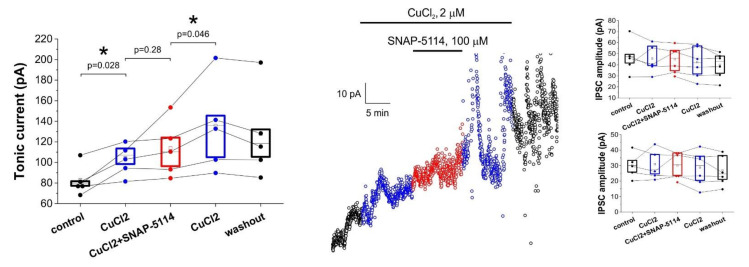
Added CuCl_2_ induces tonic inhibition which can be reduced by blocking glial GABA transporters in low-[Mg^2+^] ACSF. *Left:* Box-chart representation of GABA*ergic* baselines during control condition, CuCl_2_ application, addition of SNAP-5114 and washout. Box edges represent 25th, 50th and 75th percentile, open squares represent means, circles connected by lines represent paired individual baseline values. (N = 5). *Middle:* baseline currents plotted at 1 s intervals in a selected experiment. Large fluctuations in baseline current after SNAP-5114 washout correspond to seizure-like events. *Right*: amplitude and frequency of inhibitory postsynaptic currents (IPSCs). Box edges represent 25th, 50th and 75th percentile, open squares represent means, circles connected by lines represent paired individual baseline values. (N = 5). Neither amplitude, nor frequency changed significantly due to SNAP-5114 application (*p* = 0.81 and *p* = 0.82, respectively). Black symbols represent control and washout periods, blue symbols represent CuCl_2_ application, red symbols represent simultaneous SNAP-5114 and CuCl_2_ application in all figures.

We have shown above that inhibition of astrocytic copper uptake reduces the GAT-2/3 mediated tonic inhibitory current component, likely due to the reduced astrocytic GABA formation from putrescine. Next, we explored whether the tonic inhibitory current can be directly induced by stimulating this pathway (Figure 3). We added 2 µM CuCl_2_ to trigger copper uptake. Copper application did induce a significant increase in the baseline current measured on CA1 neurons (83.1 ± 6.7 pA in control vs. 104.2 ± 6.7 pA, *p* = 0.28, N = 5), suggesting that exogenous copper increases the astrocytic GABA level. We also demonstrated that the observed increase in the tonic current is not due to a direct effect of copper on either GABA receptors, extracellular GABA level or voltage-gated Ca^2+^ channels, since IPSC frequency and amplitude were not affected by CuCl_2_ application (Figure 3). Blockade of astrocytic GAT-2/3 transporters in this condition did not decrease the tonic current, but significantly reduced the copper-initiated increase, which continued to develop after the removal of SNAP-5114 (Figure 3). The ability of SNAP-5114 to mitigate the exogenous copper-induced enhancement of tonic current suggests that GAT-2/3 transporters are involved in the resulting GABA release. However, its inability to completely block the tonic current indicates that other efflux pathways, for example *via* the Bestrophin 1 channel [21] also needs to be considered. 

## 4. Discussion

We investigated the relationship between copper uptake, PA metabolism and subsequent astrocytic regulation of neuronal excitability. First, we reproduced our previous observation that blockade of the astroglial GAT-2/3 transporter leads to the decrease of tonic inhibitory currents in low-[Mg^2+^] activated rat hippocampal slices (*control*). Under this condition, inhibition of CTR1 by added Ag^+^ (3.6 µM AgNO_3_) eliminated the appearance of GAT-2/3 mediated tonic inhibitory currents. Since the active moiety, astrocytic GABA that mediates tonic inhibition is synthesized from putrescine, our results highlight the contribution of Cu^+^/Cu^2+^ ratio to oxidative putrescine → GABA catabolism in astrocytes [15,21,25,26,28,29,33,34,36,70]. 

Another potential pathway to discuss is the activity-dependent release of Zn^2+^ [71,72], co-released with Glu [73]. The preferential binding of Zn^2+^ to astroglial GAT-3 versus GAT-2 [74] was explained by the difference in the extracellular coordination of Zn^2+^ [75,76]. In hGAT-3, the coordination of Zn^2+^ by the extracellular EL2 loop residues (Asn190, Tyr191, Ser192) combined with residues located at the tip of the TM7 helix (Phe358, Met359, Tyr361) suggests that the blockade takes place in the outward facing open conformation of GAT-3 [76]. We expect that Cu^2+^ or Mn^2+^ may substitute Zn^2+^ in the function of stabilizing the extracellular GAT-3 gate in the open conformation by shuffling sulphur lone-pair and aromatic π electrons (MetAro effect) [77,78]. Stabilization of the open GAT-3 conformation by Cu^2+^/Mn^2+^ can also be explained by a similar MetAro effect. Since driving force of astrocytic GABA favours GABA release in the low-[Mg^2+^] medium, constant opening of GAT-3 may increase tonic inhibitory currents that were shown to be inhibited by SNAP-5114. In fact, elevated extracellular concentration of GABA was measured in the striatum of Mn^2+^-exposed rats, suggesting impairment of clearance of or enhancement of release of GABA by GABA transporters [79]. In principle, the displacement of Zn^2+^ by Cu^2+^/Mn^2+^ might also occur at other tripartite synapse targets including various Glu and GABA receptor and transporter subtypes [80,81,82,83,84,85,86,87]. These clues, however, are not likely to affect tonic inhibition via the astroglial Glu-GABA exchange mechanism, therefore they would not be sensitive to SNAP-5114.

Unexpectedly, tonic inhibitory currents elicited by ten-minute single application of 2 µM Cu^2+^ significantly exceeds those recorded with added 20 µM Mn^2+^ alone or in combination with the GAT-2/3 specific inhibitor SNAP-5114. Furthermore, the significantly higher level of tonic inhibition persists after washout, suggesting that added Cu^2+^ elicits a long-lasting shift of neuronal excitability. We propose that the tonic inhibitory currents enhancement by 2 µM CuCl_2_—that substantially exceeds the 20 µM MnCl_2_ –induced GAT-3 regulated tonic inhibitory currents—can be traced back to some additional GABA release mechanisms. At first approximation, we focused on data linked to PA metabolism. Literature data show that added Mn^2+^ dose-dependently (1–100 µM) increases putrescine content (3.2–4.5 nmol/mg protein) in human SH-SY5Y cells [88]. Besides, the increase of putrescine content positively correlates with the PA metabolite N-acetylspermidine, but negatively with GABA related metabolites [88]. It is plausible therefore, that the Cu^2+^ induced enhancement of putrescine → GABA catabolism also boosts the putrescine → spermidine → spermine metabolism pathway. Spermine may in turn support astroglial GJC coupling [30,31], long-range GABA trafficking via Cx43 GJCs and release of GABA through purinergic P2X_7_ receptor pore [89] or via connexon hemichannels like Glu [90]. These alternative GABA release mechanisms, independent of GAT-2/3 mediated gliotransmission remains to be explored in the future.

**Figure 4:**
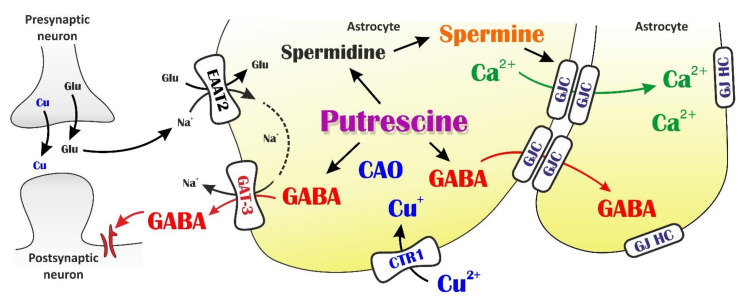
Schematic representation of dual role for astroglial copper-assisted polyamine metabolism in controlling neuronal excitability. Astrocytic putrescine can be catabolised to GABA and this GABA can release to the extrasynaptic space in response to Glu transporter (EAAT)-mediated Na^+^ influx. The released GABA generate tonic current on extrasynaptic GABAa receptors. Since the putrescine-GABA conversion is catalyzed by copper containing amine oxidases (CAOs), intracellular copper level can influence the amount of GABA accumulated in astrocytes. By inhibiting or stimulating the astrocytic copper transporter CTR1, the releasable GABA pool can be controlled. On the other hand, putrescine is also metabolized to spermine which contributes to opening of gap junction channels (GJCs), through which Ca^2+^ and other substances can be redistributed through the astrocytic syncytium and consequently neuronal activity is spread over a large area.

Data on depolarization-induced bulk copper release amounts to ≥ 100 μM synaptic transients (*for a detailed discussion see* [41]). Synaptic copper transients spread over the extra-synaptic compartment by a steep ≥ 100 μM (synaptic) → ≤ 1 μM (extra-synaptic) copper gradient [91,92]. The *K_m_* values characterizing copper uptake by CTR1 range between 1 μM and 6 μM [44]. These findings explain the efficient extra-cellular modulation of astroglial CTR1 by adding 2 µM CuCl_2_. Our data provide supporting evidence on the gliocentric scheme of copper signaling [41] by relating astroglial copper uptake to feedback inhibition of neuronal excitability. Multiple mechanisms comprising several steps can be identified to interpret these data (Figure 4):

(1) Uptake of extrasynaptic Cu^2+^ is mediated by the astroglial CTR1 that provides copper for CAO. This pathway can be blocked by Ag^+^. (2) Changes in intracellular copper concentration affect CAO activity. Although copper loading into the CAO active site is considered to be an irreversible process, the rate by which the loading occurs does depend on solute copper concentration. An experimentally validated kinetic model developed by Adelson et al. [93] showed that 12 h loading with 0.8 equiv. Cu^2+^ is comparable with 30 min loading with 10 fold copper excess [94]. The dependency of copper loading time on intracellular copper level is likely to be attributed to rapid (> 0.1 s^−1^) copper binding to a second, reversible, pre-equilibrium “kinetic” site on CAO [93]. It was also directly shown that copper level does influence CAO activity [95,96]. (3) Copper assisted putrescine catabolism to GABA provides elevated intracellular GABA and enhanced GABA release. (4) GABA release through outward open GAT-3 triggers the enhancement of tonic inhibitory currents via activation of the extrasynaptic GABA_a_ receptor subtype. (5) Cu^2+^ may also bind to the extracellular Zn^2+^ binding sites of the GAT-3 transporter, keeping GAT-3 open and therefore facilitating GABA release. (6) On the other hand, putrescine is also used to synthesize spermine which keeps gap junction channels (GJCs) open and enables inter-cellular trafficking of Ca^2+^ and other messengers. (7) Alternatively, GABA can also spread through the GJCs and released at distant astroglial processes through P2X_7_ purinergic receptor pores or Cx43 hemichannels. 

Consequently, astrocytic putrescine metabolism simultaneously participates both in reduction and enhancement of neuronal activity by providing tonic GABA currents and GJC-mediated activity-spreading, respectively.

The amount of copper released into the synapse, diffused extra-synaptically and taken up by astroglial copper transporters determines the strength of redox coupling which may vary from physiological to up- or down-regulated. The high extracellular level of copper elicits low surface expression of CTR1 [44], that may be mechanistically linked to Parkinson’s and Alzheimer’s disease mechanisms [97,98] or schizophrenia [99]. Metabolic profiling of brains [100,101,102] and serum [103] of AD subjects suggests a possible role for altered spermidine metabolism correlating AD indicators. Further studies may also substantiate spermidine-associated change of putrescine and GABA that can serve as early serum biomarkers for AD progression in the future. 

## Data Availability

The data supporting this manuscript is available upon request to the corresponding author (László Héja, heja.laszlo@ttk.hu).
